# Development and Characterization of Meat-Based Pasta Enriched with Apple and Sugar Beet Fibers

**DOI:** 10.3390/foods14223837

**Published:** 2025-11-09

**Authors:** Diana-Remina Manoliu, Mihai Cătălin Ciobotaru, Marius-Mihai Ciobanu, Paul-Corneliu Boișteanu

**Affiliations:** 1 Faculty of Food and Animal Sciences, “Ion Ionescu de la Brad” Iasi University of Life Sciences, 8 Mihail Sadoveanu Alley, 700489 Iasi, Romania; diana.manoliu@iuls.ro (D.-R.M.); paul.boisteanu@iuls.ro (P.-C.B.); 2 Faculty of Agriculture, “Ion Ionescu de la Brad” Iasi University of Life Sciences, 3 Mihail Sadoveanu Alley, 700490 Iasi, Romania

**Keywords:** meat-based pasta, dietary fibers enrichment, novel food development, food technology, functional ingredients in meat products, consumer perception

## Abstract

The global trend toward sustainable and health-promoting foods has encouraged the reformulation of meat products that strategically incorporate high-quality animal proteins and functional compounds derived from plants. This study focuses on a complex food concept: meat-based pasta formulated from pork, semolina, and dietary fibers (apple and sugar beet). The quality attributes and the effects of different formulations were evaluated in comparison with a control sample. The findings show that the addition of dietary fibers significantly impacted the chemical composition, lowered the pH and increased water activity. The incorporation of the apple and sugar beet fibers increased the total dietary fiber content from 2.94% (control) to 9.59% and 11.15%, respectively, at the highest level of inclusion. Moreover, texture profile analysis of the raw samples revealed an enhancement in hardness (from 8.01 N in the control to maximum values of 21.23 N and 26.37 N), gumminess (from 3.28 N to 10.43 N and 12.36 N), and slight improvements in cohesiveness (from 0.41 to maximum values of 0.49 and 0.51) with the addition of apple and sugar beet fibers, respectively. The color parameters (L*, a*, b*) varied depending on the fiber source, with beet fiber imparting higher lightness and redness, while apple fiber contributed to darker tones. An initial consumer acceptability test revealed a positive perception of the innovative product, particularly for formulations with low and medium percentages of fiber addition. Overall, the results demonstrate that meat-based pasta can be successfully formulated with dietary fibers, providing an innovative and feasible alternative that meets current consumer expectations for novel, healthy, and sustainable foods.

## 1. Introduction

The food industry has shown a growing interest in developing healthier, high-quality foods produced through minimal processing. With increasing concerns about the health risks associated with highly processed products, consumer demand for “eco-friendly”, “organic”, and “natural” foods has risen sharply. In response, the meat industry is focusing on alternatives to synthetic additives [1,2].

Nutrition is now recognized as a critical determinant of human health and well-being, and public awareness of this link continues to expand. The rising prevalence of lifestyle-related diseases has created a strong demand for health-promoting or functional foods designed to improve health outcomes and reduce the risk of diet-related disorders [3]. As a result, consumers increasingly seek meat products with reduced levels of salt, fat, cholesterol, nitrites, and calories [4], while also showing interest in formulations enriched with bioactive compounds such as carotenoids, unsaturated fatty acids, sterols, and dietary fiber [5,6]. Although meat is a valuable source of high-quality proteins containing nearly all essential amino acids, it remains a poor source of dietary fiber [6,7]. According to the Food and Drug Administration [8], the recommended daily intake of dietary fiber is 28 g. However, due to shifts in eating habits, unbalanced diets, and a lack of moderation and variety, much of the population fails to meet this requirement.

According to the Codex Alimentarius Commission, claims regarding high content and source of dietary fiber are defined based on reference amounts per serving, 100 g, and 100 kcal. European legislation states that to claim that a food is a “source of fiber”, it must contain at least 3 g of fiber per 100 g (or 1.5 g of fiber per 100 kcal). To claim that a food is “high in fiber”, it should have at least 6 g per 100 g (or 3 g of fiber per 100 kcal) [9]. For example, the dietary fiber content of white bread, determined by a standardized method (AOAC 2009.01), is 4.1 g/100 g, which would allow the claim “source of fiber” [10,11,12].

In this context, there is a growing trend toward enriching meat products by incorporating ingredients of non-animal origin. Such an approach offers several advantages, including: (1) flavor and acceptability: adding non-animal ingredients can intensify flavor notes and increase overall consumer acceptability [13]; (2) moisture retention: proteins derived from soy or dairy, as well as carbohydrates such as starch and carrageenan, contribute to effective moisture retention [14,15]; (3) freeze–thaw stability: the use of modified starch improves product stability during freezing and thawing; (4) texture and consistency: the gelatinization of soy protein can modify and improve the texture of the final product [16]; (5) visual appearance: ingredients such as paprika or beetroot powder impart attractive color attributes to the product [17]; (6) economic efficiency: the integration of non-animal ingredients can reduce formulation costs and increase product yield [18]; (7) handling and storage: ingredients such as carrageenan supports gel formation that improves shear stability, while other compounds, such as lactic acid or spice extracts, can extend product shelf life.

Overall, the integration of non-animal ingredients into meat products not only enhances quality and functionality but also provides additional benefits in terms of cost-effectiveness and consumer satisfaction [14,19].

Dietary fiber imparts important functional properties to foods, including enhanced water- and oil-holding capacities, emulsification, and gel formation. When incorporated into a variety of products such as baked goods, dairy items, jams, meat products, or soups, dietary fiber can alter textural attributes, prevent syneresis (liquid separation due to gel contraction), stabilize high-fat systems and emulsions, and improve shelf life [20].

Nevertheless, the cooking method significantly influences the quality of fiber-enriched meat products, particularly in terms of moisture and fiber retention [21]. Smoking contributes to product stabilization through its antimicrobial and antioxidant activities, ensuring an extended shelf life while imparting distinct sensory characteristics [22]. In line with this, Choi et al. [23] reported that adding chicory fiber as a partial fat substitute, combined with smoking treatment, can contribute to the development of restructured meat products with functional value, maintaining quality characteristics by compensating for the negative effects of fat replacement on physicochemical and sensory parameters. Similarly, steam cooking of fiber-enriched chicken nuggets maximizes cooking yield and retains fiber and moisture as steam reduces the rate of water loss during processing, thereby ensuring higher product juiciness [21].

In the food sector, and particularly in the meat industry, there is a need to develop new and complete products, as consumers tend to seek nutritionally enhanced functional meat products [24]. Research in the food sector shows numerous attempts to fortify traditional meat products with different sources of vegetable fiber in order to obtain functional products with improved nutritional value. However, previous studies on fiber-enriched meat products have mostly focused on traditional comminuted formulations such as sausages, patties, meatballs or burgers, while some addressed the formulation of meat noodles, and not the complex fresh meat pasta matrices with fiber enrichment.

By enriching meat with dietary fiber, manufacturers can create foods that combine high-quality protein with fiber, thereby improving the nutritional profile of the final product. Beyond their nutritional advantages, fiber-enriched meat products represent a promising category of complex foods that deliver multiple benefits for consumer health.

In addition to nutritional benefits, the incorporation of dietary fibers induces a range of physical modifications that affect the technological performance of products during processing, as well as sensory changes that may alter consumer perceptions and expectations when compared with familiar conventional products [25]. Moreover, dietary fibers can serve as partial substitutes for artificial additives due to their functional properties, including textural stabilization [26], water-binding capacity [27] and oil absorption [20,28]. These applications can improve consumer perception, especially in the context of growing concerns about the risks associated with synthetic substances and the increasing demand for natural additives and clean-label foods [29].

Apple and sugar beet fibers were selected as enrichment sources due to their abundance in European agro-industrial sectors [30,31], with apple production representing approximately 13% of global output (11.01 million metric tons in 2024/2025) according to the United States Department of Agriculture [32], and EU beet production reaching 15.3 million tonnes as reported by European Association of Sugar Manufacturers [33]. Moreover, their hydration and swelling capacities, along with their impact on textural reinforcement when incorporated into meat systems, provide functional advantages in the formulation of meat products [34,35]. The use of these fibers contributes to up-cycling of fruit and sugar industry, while addressing nutritional enhancement and sustainability demands within the modern meat sector [36,37]. As shown by Obidziński et al. [38], apple fiber is a by-product from apple processing industry, characterized by a high content of soluble dietary fibers, such as pectins, organic acids, vitamins and tannins. These properties confer potential for improving the nutritional profile of reformulated products and are relevant in the context of the sustainable valorization of food industry by-product streams. Sugar beet fiber is also an agro-industrial co-product with a high water-holding capacity and valuable texturizing effects, which makes it suitable for enriching meat products and improving their technological properties [35]. As demonstrated by studies applied to meat products [39], as well as to other categories of food products such as sponge cakes [40], the incorporation of sugar beet fiber contributes to increased water retention, enhanced structural stability, and optimization of the final product’s texture.

The development and promotion of these products can also stimulate innovation in the food industry, promoting diversification of the range of products available and encouraging the consumption of healthy foods. In this regard, the role of mass media, as a key factor in promoting education, performance, and behavioral change [41], can be equally important in shaping consumer attitudes toward innovative food products. In an era of growing concern for health and sustainability, effectively communicating the benefits of dietary fibers and their contribution to the diet through media platforms can enhance public understanding and acceptance of reformulated meat products. Such awareness and consumer engagement are essential to encourage behavioral change and support the development of healthier diets; consequently, new meat products enriched with dietary fiber can play an important role in promoting a healthy and balanced lifestyle.

Within this framework, the present research aimed to formulate meat-based pasta enriched with apple and sugar beet fibers, to characterize the variants obtained, and to evaluate the influence of these additions on the physicochemical and sensory quality of the products.

## 2. Materials and Methods

### 2.1. Materials

Fresh pork leg meat, semolina flour, apple and sugar beet fiber, salt, beetroot powder, black pepper, onion powder, and bone broth used in the preparation of meat-based pasta were food grade and acquired from either local market or commercial food suppliers. Specifically, pork leg meat was purchased from a local slaughterhouse in the northeastern region of Romania (Sagrod SRL), while the fibers and spices were purchased from a commercial supplier (Rocas FDS).

### 2.2. Samples Preparation

The pork meat was stored under refrigeration at 0–4 °C until processing. Prior to use, visible fat and connective tissue were removed, and the meat was cut into small pieces to facilitate grinding. The prepared pork meat was ground using the grinder (Grinder WP-105) with 5 mm diameter sieve, followed by fine comminution in a silent cutter (Cutter Titane V 45L). As shown in Table 1, seven experimental formulations of fresh meat-based pasta were prepared by partially replacing semolina flour with two types of fibers in three inclusion levels: apple fiber (3%, 6%, and 10%), and beet fiber (3%, 6%, and 10%). A control sample (Con) was produced from pork meat and semolina flour without fiber addition. The mixing ratio of pork meat to semolina was determined in preliminary trials to ensure optimal dough formation.

After obtaining the meat batter, flour, fiber and spices (black pepper, onion powder, beetroot powder) were incorporated into the composition and kneaded until a uniform dough consistency was obtained. The beetroot powder was incorporated into the standardized spice mix at a low and constant level across all formulations to enhance the characteristic reddish appearance of meat-based pasta products and ensure uniform visual appeal, without influencing comparative color differences among treatments. During this technological process, the temperature of the mixture was carefully monitored and maintained below 10 °C to ensure proper dough consistency and to minimize microbiological risks. The final dough was formed into a pasta shape using a pasta machine (Sansone 42XP, Sirman Spa, Pieve di Curtarolo, Italy). Drying was carried out in an industrial chamber (INDU iMAX500 heat treatment chamber) at a temperature of 35 °C and a set humidity of 10% for 30 min, to ensure the characteristic texture and moisture associated with fresh pasta.

### 2.3. Physical and Chemical Analyses

The proximate composition of the raw samples was determined using standard analytical methods of the Association of Official Agricultural Chemists (AOAC) as described by Naghdi et al. [42]. Moisture content was assessed using the oven-drying method, crude lipid content was determined by Soxhlet extraction, crude protein content was measured using the Kjeldahl method, and ash content was measured using the dry-ashing method. Total fiber content was quantified using an enzymatic–gravimetric procedure (AOAC 985.29) as outlined by Bajcic et al. [43]. The total available carbohydrate content was calculated by difference, according to Equation (1), provided by Menezes et al. [44]:

Carbohydrate content (%) = 100 − (moisture + fat + ash + protein + dietary fiber)(1)

The total energy value was calculated by multiplying the percentage of carbohydrate content by 4 kcal/g, protein content by 4 kcal/g, fat content by 9 kcal/g and fiber by 2 kcal/g [45]. Energy was expressed as kilocalories per 100 g (kcal/100 g).

The acidity of meat dough was determined using the direct method, with pH measured by a digital pH meter (HI98163, Hanna Instruments, Nusfalau, Romania) specifically designed for meat. Prior to analysis, the pH meter was calibrated with two buffer solutions (pH 4.01 and pH 7.01); after equilibration, the electrode was inserted directly into the sample, and the pH value was recorded once the device stabilized.

Water activity (a_w_) was determined using an AquaLab 4TE instrument (Addium Inc., Washington, DC, USA). Prior to analysis, samples were ground to ensure uniform moisture distribution.

### 2.4. Instrumental Analyses

Color measurements were performed on dried pasta samples because drying stabilizes surface pigmentation and reduces moisture-induced gloss variation, resulting in more reliable CIELAB readings. In contrast, textural properties were evaluated on both raw and cooked samples to capture mechanical differences induced by processing, hydration, and structural matrix development.

The instrumental determination of color was performed using a portable Chroma Meter CR-410 colorimeter (Konica Minolta Inc., Tokyo, Japan), applying the CIELAB system to measure brightness (L*), red–green (a*) and yellow–blue (b*) coordinates, with a D65 illuminant, and a light aperture size of 50 mm.

The Chroma value (C*) represents the color saturation, indicating the distance from the central achromatic gray axis of the color space, while the hue angle (H*) reflects chromaticity or color tone, ranging from 0° (red), 90° (yellowish), 180° (green), and 270° (blue) to 360° (red). The conversion from CIE Lab to CIE LCH was performed using Equations (2) and (3) [46,47]:

(2) $$Hue\;angle\;\left(H^{*}\right)=\;tan^{-1}\times b^{*}/\;a^{*}$$

(3) $$Chroma\;\left(C^{*}\right)=\sqrt{{\left(a^{*}\right)}^{2}+{\left(b^{*}\right)}^{2}}$$

The texture of meat pasta samples was evaluated using compression and shear tests, performed on a Mark-10 texturometer equipped with a series 5 dynamometer with a maximum measurement capacity of 250 N and a resolution of 0.02 N. Prior to testing, all samples were conditioned at room temperature (20 ± 1 °C) for 30 min to minimize temperature-related variability. Shear tests were performed with a Warner–Bratzler probe, and the data were recorded as force-time and force-displacement curves. For shear force measurements, the samples were prepared from pasta dough formed into uniform compact cylinders (10 cm in length and 15 mm in diameter), and three replicates were recorded for each batch. The parameters determined included cutting force (N) and work of cutting (mJ).

For texture profile analysis (TPA), compression tests were performed with a cylindrical probe, at a crosshead speed of 200 mm/min and a compression depth of 1 cm, involving two successive compressions. Samples were prepared into cylinders of 22 mm in diameter and 30 mm in length, ensuring consistent geometry across treatments. Results were obtained using the MesurGauge+ program, which generated force-time and force-displacement curves. Following compression, textural profile parameters were determined, including hardness (maximum force at first compression), cohesiveness, elasticity, gumminess, and adhesiveness.

### 2.5. Sensory Perception and Evaluation

The sensory evaluation of meat pasta batches enriched with dietary fibers was conducted with a group of 30 panelists, aged between 22 and 42 years, that were selected based on their regular consumption of pasta and meat products (≥2 times/month), absence of sensory impairments or food allergies related to the tested ingredients and willingness to participate in two evaluation sessions. The evaluation session was organized in a specialized sensory laboratory, with individual booths, under white light, and at a controlled temperature, using discriminative and descriptive methods. Prior to testing, panelists received a short briefing to present the product, to familiarize them with the evaluation procedure, attribute definitions, and use of the scoring scale.

The sensory evaluation was structured in two stages: the first stage consisted of a brainstorming session aimed at identifying the most relevant sensory terms and attributes specific to the product category under study, followed by a second stage, which involved the actual evaluation using a questionnaire that summarized the list of attributes generated in the first stage.

The questionnaire included two types of sensory tests: the Check-all-that-apply (CATA) test and Quantitative Descriptive Analysis (QDA). The combination of CATA and QDA was chosen to integrate both consumer-based perception and intensity-based descriptive profiling. The CATA test assessed the overall perception of the evaluators and served as an effective method for recording the presence or absence of sensory attributes, as evaluators selected all attributes they identified in each sample without assigning intensity scores [48], while QDA quantified the intensity of each sensory attribute on a 9-point scale (1 = extremely dislike; 5 = neither pleasant nor unpleasant, 9 = extremely like) for all product batches.

The evaluators received a predefined set of descriptive attributes along with pasta samples to evaluate (Table 2). The samples were displayed in a randomized order to prevent mutual influence among evaluators.

### 2.6. Statistical Analysis

All analyses were performed in triplicate, and the results were presented as mean value ± standard deviation (SD). Statistical analysis of the data obtained was carried out using SPSS (IBM SPSS Statistics V21) and XLSTAT software. IBM SPSS Statistics was used to evaluate significant differences between variants and to analyze relationships between sensory variables by applying statistical methods such as ANOVA (Analysis of Variance) and Tukey’s test, while XLSTAT was used to centralize sensory analysis results and to generate symmetric plot graphs and principal coordinate analysis (PCoA).

## 3. Results and Discussion

### 3.1. Physico-Chemical Characterization of Meat Pasta Samples

Meat-based pasta represents a complex fresh product, with advantages such as high nutritional potential, versatility and convenience in preparation. The proximate composition of the control and fiber-enriched pasta samples, formulated with apple and sugar beet fibers, is presented in Table 3. Similar to other functional food systems, the addition of plant-derived ingredients can modify the chemical composition of the final product by altering moisture, protein, fat and dietary fiber contents.

The incorporation of apple and sugar beet fibers significantly (*p* < 0.05) influenced the nutritional profile of the products in terms of moisture, fat, protein, ash, fiber, carbohydrates and energetic value. A higher fat content was observed in all experimental formulations compared with the control, whereas the total dietary fiber content increased proportionally with the level of substitution. Moisture values showed slight variations, reflecting the high water-binding capacity of the added fibers.

The effect of fiber addition was a reduction in dry matter at the first two addition levels (3% and 6%), followed by a slight increase in the 10% variants. This trend was observed in both formulations (apple fiber and sugar beet fiber) compared to the control sample.

In addition, incorporation of vegetable fibers led to an increase in water content, with varying intensity depending on fiber type and concentration, compared to the control sample, which had the lowest water content (25.20 ± 0.16%). The added fibers contributed significantly to moisture retention in the enriched variants, particularly during the drying process, acting as water retention agents due to their high capacity to bind free water within the food matrix [49,50]. Similar results were reported by Boișteanu et al. [51], who found that enriching beef burgers with red lentil flour (5 and 10%) improved moisture retention in the supplemented sample. Choi et al. [52] also observed the same effect of increased moisture at lower inclusion levels (1% and 2%) of apple pomace fiber.

The moisture retention effect is attributed to the polysaccharide structure of the fibers and their functional properties (water-holding capacity, gel formation, viscosity increase). Moreover, water, as a dipolar molecule, is attracted to charged substances such as proteins, with myofibrillar proteins being primarily responsible for the high-water retention capacity. During the pasta preparation, the flour incorporation leads to hydration of starch granules, around which a protein film forms. Meanwhile, the incorporation of meat emulsions increases the amount of charged proteins, thereby improving water retention and elevating moisture levels [49,53].

Fat content data show that fiber addition influenced this parameter differently, depending on the type of formulation. The control batch had a fat content of 2.06 ± 0.10%, reflecting the natural fat level of the product matrix, which consists mainly of pork leg meat and semolina flour [54,55]. With fiber addition, notable variations in fat content were observed depending on fiber type and concentration.

Apple fiber exerted the most pronounced effect, with fat content increasing slightly to 2.23 ± 0.10% at 3% inclusion and reaching 2.78 ± 0.12% at 10%. This trend suggests that apple fiber improves fat retention, possibly due to its structural properties that facilitate the incorporation of fat-soluble components into the matrix [56]. These findings highlight the potential of plant fibers, particularly apple fiber, to improve fat retention in meat pasta formulations. Sugar beet fiber showed a more moderate impact, with fat content increasing to a maximum of 2.30 ± 0.10% at 10% inclusion, reflecting its intermediate ability to bind and stabilize fat [39].

Protein content was also significantly affected (*p* < 0.05) by the addition of dietary fibers, with reductions observed in the enriched formulations. The control batch maintained the highest protein content (23.82 ± 0.13%), reflecting the natural protein level of pork [57]. This value serves as a basis for comparison with fiber-enriched variants, where the protein content is influenced by both type and concentration of fiber.

Generally, fiber addition led to a reduction in protein content, to a minimum value of 19.68 ± 0.11% recorded at 6% sugar beet fiber inclusion. This reduction is likely due to the water-binding capacity of fibers, which may interfere with protein retention in the product. Polysaccharides can interact with myofibrillar proteins through hydrogen bonding, electrostatic interactions, and steric hindrance, potentially affecting protein retention during processing [58]. Furthermore, the formation of fiber–protein networks may decrease protein extractability due to hydrogen bonding and electrostatic interactions [59,60], which can lead to an apparent reduction in measured protein content.

However, at the 10% inclusion, a slight recovery in protein content is observed across all formulations, possibly due to improved interactions between fiber and the protein matrix during processing. Parkash et al. [61] similarly reported a decrease in protein content with the addition of 6% dried apple pomace in chevon rolls, from 18.78% in the control sample to 16.75% in the enriched variant.

The ash content was significantly influenced (*p* < 0.05) by the fiber addition. Within formulations containing the same fiber type, smaller statistical differences were observed. In the apple fiber variants, sample AF-6 was significantly different, with the lowest ash content (4.22 ± 0.03%), compared to the other addition levels.

Overall, enrichment with apple fibers led to reduced ash content at all levels compared to the control (4.88 ± 0.04%). In contrast, although initially following a similar trend (ash decreased to 4.66 ± 0.03% and 4.69 ± 0.01% at 3% and 6%, respectively), beet fibers cause an increase in ash content to 5.02 ± 0.03% at the highest addition level. The increase in ash content observed in BF-10 can be attributed to the intrinsic mineral content of sugar beet fiber of 5.31–5.63%, as reported by Vural et al. [39] and Zachesova et al. [49]. Furthermore, soluble dietary fiber derived from sugar beet pulp can retain mineral constituents within the polysaccharide matrix [62]. Data reported for other fiber-enriched meat products show that similar increases in ash content have been observed in beef burgers enriched with orange albedo flour (from 2.59% in control sample to 3.20% at the highest inclusion level) [60]. Likewise, other studies reported similar results at the inclusion of pomegranate peel, date paste or date pulp in different meat products (chicken meat patties, chicken meat nuggets, bologna sausages, camel meat burger) [63,64,65,66,67].

Fiber addition also significantly affected the fiber and carbohydrate content of meat pasta formulations, improving their nutritional profile. The control batch, without added fiber, had a total fiber content of approximately 2.94 ± 0.01%, reflecting the natural content of durum wheat flour [68].

With apple and sugar beet fiber inclusion, total fiber content increased significantly (*p* < 0.05). Apple fiber contributed to a steady increase in fiber levels as its inclusion increased, reaching 11.15 ± 0.06% at 10%, while sugar beet fiber follows the same trend, with the fiber content in meat pasta samples increasing to 9.59 ± 0.08% in variant BF-10. These results are consistent with previous studies [20,56,61,69,70], confirming the substantial contribution of apple and sugar beet fibers to total dietary fiber levels in enriched food matrices.

Conversely, the fiber addition reduces the carbohydrate content in the six experimental formulations compared to the control, with the reduction intensity varying according to fiber type and inclusion level. The control batch had the highest carbohydrate content (41.11 ± 0.15%), attributed to semolina flour, which is rich in digestible carbohydrates [68]. With apple fiber inclusion, the carbohydrate levels decreased significantly, reaching 29.63 ± 0.20% at 10% substitution. Sugar beet fibers have a comparable effect, reducing the carbohydrate content to 30.00 ± 0.13% at the same inclusion level.

These findings are similar with Elleuch et al. [20], who reported that fiber addition reduces available carbohydrate content by replacing digestible carbohydrate sources with indigestible polysaccharides. Similar trends were reported by Ktenioudaki et al. [71] and Wee & Henry [72], who observed the varied effects of dietary fiber integration on reducing digestible carbohydrate levels.

### 3.2. Determination of pH and Water Activity

**Figure 1:**
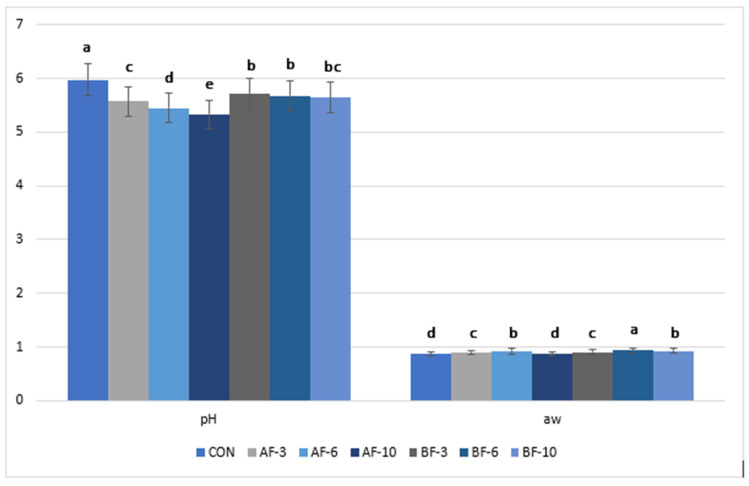
Change in pH and water activity (a_w_) in different variants of meat pasta (different letters above the bars indicate significant differences between samples, *p* < 0.05).

The pH of meat pasta samples with different plant fiber additives was determined on the dough obtained prior to drying. The average pH values (Figure 1) for the seven samples analyzed ranged from 5.33 ± 0.04 to 5.97 ± 0.03, with the maximum pH value recorded in the control sample. A significant decrease in pH was observed in the enriched formulations, which intensified with increasing inclusion levels. In the case of apple fiber addition, the pH-lowering effect may be associated with the intrinsic acidity of apple powders, with the literature reporting the presence of organic acids, particularly malic acid, which lowers pH [73]. In combination with the pH of pork and semolina flour, this resulted in a lower pH for the fiber-enriched pasta, compared to the other variants. These findings are consistent with those of Younis & Ahmad [56], who reported a decrease in pH in buffalo meat sausage incorporated with 6% apple pomace powder.

The sugar beet fiber variants showed a less pronounced acidifying effect compared with apple fiber, with the lowest pH recorded in the 10% sample, 5.64 ± 0.03. Zarei & Nateghi [74] also reported a significant decrease in pH in red grape juice samples following the addition of mixtures of sugar beet and inulin fibers.

The introduction of vegetable fibers—apple, beet—into meat pasta formulations significantly influenced water activity (a_w_), an essential parameter in microbiological stability and shelf life. The control pasta, without added vegetable fiber, recorded a water activity of 0.877 ± 0.01 (Figure 1), an intermediate value considered normal for a complex product of this type, given that the water activity in fresh meat products ranges between 0.98–1.00, dried meat products between 0.80–0.92, and fresh pasta between 0.92–0.94 [75,76].

At inclusion levels of 3% and 6%, water activity increased significantly compared with the control sample. However, at 10% addition level, water activity decreased in the pasta formulations. Similar findings were reported by Kerner et al. [77], who observed an increase in water activity in pork burger patties formulated with sweet grass extract and dried pressed hemp seedcake. In line with these results, Younis & Ahmad [56] reported an increase in water activity from 0.97 in the control buffalo meat sausage to 0.99 in the variant containing 6% apple pomace powder. The differences in a_w_ can be explained by the water-binding properties of fibers and their interactions with the meat matrix at different inclusion levels. This water activity behavior results from fiber–water interactions, including hydrophilic polysaccharides (e.g., pectin), and also proteins, which can immobilize free water through hydrogen bonding, capillary retention, and physical entrapment within the fiber matrix, increasing the proportion of immobilized water in the system [78]. Similar findings were reported by Zhu et al. [79], who demonstrated that hydrophilic compounds reduced the rate of aw decrease during storage, after an initial increase in water activity from 0.972 to 0.984, by altering the gluten network and limiting moisture migration. However, at excessive addition levels, fiber may disrupt the structural continuity of the matrix, creating preferential pathways for water release and subsequently decreasing aw, consistent with the reduction observed at 10% inclusion.

### 3.3. Colorimetric Analysis

The color of food is important in initial perception, as it provides both visual appeal and information on potential chemical changes during processing. The incorporation of apple and beet fibers significantly (*p* < 0.05) affected the color parameters of meat pasta after drying (Table 4).

Apple fiber significantly reduced (*p* < 0.05) lightness (L*), particularly at 10% inclusion (AF-10: 36.46 ± 2.45), making the meat pasta appear visibly darker than the control sample prepared only with meat and semolina flour (42.68 ± 1.72). In addition, apple fiber decreased the yellow hue (b*), compared to sugar beet fiber variants, indicating its strong ability to reduce the chromatic intensity of the product. Similarly, the red-green coordinate (a*) decreased significantly (*p* < 0.05) with increasing fiber addition. These changes, as well as the significant decreases in color saturation (C), resulted in brown color changes due to the natural compounds in apple fibers (carotenoids, pigments) and the degradation of chlorophyll during apple processing [80].

The addition of sugar beet fiber also induced significant differences compared to the control sample, although the effect of concentration was less pronounced. Lightness (L*) showed no significant variation (*p* > 0.05) between the 3%, 6%, and 10% inclusion levels. However, redness (a*), yellowness (b*) and saturation (C) values for the 10% sample were significantly different (*p* < 0.05) from those at lower concentrations.

Overall, sugar beet fiber addition increased brightness (L*), correlated with the higher intrinsic brightness of the powder used. Nevertheless, lightness values for beet fiber–enriched pasta remained within the range reported for pork leg muscle (46.13–48.53), according to Bednářová et al. [81].

The hue angle (H*) recorded a low value for the control sample, indicating a red coloration, typical for meat products, where red tones predominate. Fiber addition increased H* value, suggesting a color shift from red toward yellow-orange, likely due to the non-extractable polyphenolic compounds in plant fibers, which can interact with myoglobin and change the perceived product color [82].

Therefore, apple fibers produced darker shades with more intense red and yellow tones, while sugar beet fibers contributed to maintaining a brighter, more neutral color, closer to that of the control sample. These visual differences can impact consumer appeal and preference, as color is known to play a key role in consumers’ initial perception and acceptance of food products. Recent studies highlight that darker tones are frequently associated with natural, richer flavors and greater nutrient density, while brighter more neutral colors tend to be perceived as more familiar and acceptable [83,84].

### 3.4. Texture Analysis of Meat Pasta

The texture of meat products is a key determinant of consumer experience, and objective texture measurements, using a texturometer, provides data on the internal structure of meat pasta, which can be correlated with the chemical composition and protein quality [35]. The texture analysis followed two types of parameters: cutting parameters—Warner–Bratzler cutting force and work of cutting (mJ)—and descriptive texture parameters—hardness (N), cohesiveness, elasticity, gumminess (N), and adhesiveness—the results of which are presented in Table 5.

In the first stage, the impact of fiber type and inclusion level on Warner–Bratzler cutting force and work of cutting was evaluated for raw and cooked pasta. In the raw state, pasta without fiber addition (control) showed significantly lower shear force values, with an average value of 4.82 ± 0.68 N/cm^2^. Fiber addition generally increased shear force in most pasta formulations, except for the 3% sugar beet fiber variant, which recorded 4.27 ± 0.09 N/cm^2^, reflecting a hydration effect similar to the control sample. In contrast, at higher inclusion levels (6% and 10%), the cumulative effects of fiber binding properties, structural reinforcement, and the limited available water in the meat and in the manufacturing process led to a significant increase in shear force and work of shear, indicating a tougher structure and greater firmness [6,85].

Similar to cutting force, sugar beet fiber demonstrated the most significant impact, as work of cutting decreased below the control (36.70 ± 1.55 mJ) for the 3% addition, but peaked at 6% addition (58.84 ± 2.15 mJ). This may be explained by the effective integration of fiber into the protein matrix at moderate levels, which strengthens the structure and increases shear resistance, while excessive addition of 10% can create a denser, less cohesive structure due to possible agglomerations or uneven distribution of the fiber addition [86]. Giménez-Ribes et al. [87] also demonstrated that fibrous plant-based structures tended to break down at large strains, similarly to the behavior observed in the formulations with higher fiber inclusion levels, where excessive structuring may lead to increased cutting force and work of shearing.

The cooked control sample (without fiber addition) exhibited intermediate values for cutting force (1.20 ± 0.14 N/cm^2^) and work of cutting (18.19 ± 3.14 mJ). The addition of apple fibers caused a progressive decrease in cutting force, with a significant reduction (*p* < 0.05) observed only at the 10% level (AF-10, 0.91 ± 0.11 N/cm^2^). This effect is attributed to the high capacity of apple fibers to retain water and disperse the protein network formed during cooking [56]. As a result, the structure became more aerated and less compact, requiring less mechanical effort for cutting. Also, the presence of pectin and soluble compounds in apple fibers can interfere with protein gelation, which further contributes to reducing consistency [88].

Sugar beet fibers displayed a different cutting behavior after boiling, with higher Warner–Bratzler shear force values at 3% and 6% addition (1.32 ± 0.17 N/cm^2^ and 1.23 ± 0.20 N/cm^2^ respectively), comparable to the control. At higher concentrations (10% inclusion), a slight reduction was observed (1.17 ± 0.19 N/cm^2^), but sugar beet fiber variants maintained firm textures, indicating a stabilizing effect. This can be explained by the composition of beet fibers, rich in hemicellulose and insoluble fibers [89], which can contribute to the formation of a cohesive network, less susceptible to dilution or excessive swelling during heat treatment. These findings are in line with Giménez-Ribes et al. [87], who showed that cooked meat exhibits a marked strain-stiffening behavior and reduced energy dissipation at high deformations, reflecting a stronger, more cohesive network.

Descriptive texture parameters obtained by compression tests (Table 5) were defined as follows: hardness (N)—the force required to compress the sample, represents the resistance of the material to the compressive force; cohesiveness—the ability of the sample to remain compact during deformation; elasticity—the ability of the sample to return to its original shape after compression; gumminess (N)—a measure of cohesiveness and hardness, shows how much energy is required to compress the sample; and adhesiveness—measures the force required to resist adhesion to the surface of the compression element [90,91].

Hardness was lowest in the control sample (8.01 ± 0.42 N), as the absence of fibers does not provide additional structural support in the meat matrix. With fibers addition, a significant impact (*p* < 0.05) on hardness was observed. Apple fiber caused a steady increase in hardness, to a peak of 21.23 ± 0.57 N in sample AF-10, indicating high compressive strength due to significant reinforcement of the meat matrix after fiber addition. However, although the increase was evident for the three levels of introduction compared to the control sample, at 6% inclusion, a decrease in hardness was observed (from 14.24 N—3% to 12.68 N—6%), due to the complex interaction between fiber concentration, water retention, and fiber-protein interactions [34]. At higher levels, apple fiber absorbs and redistributes water more efficiently, strengthens the structure through gelation, and improves cohesion by stabilizing the protein network, resulting in a denser and firmer structure [56]. Similarly, sugar beet fiber exerted a stronger effect, increasing hardness from 13.74 ± 1.37 N (BF-3) to 26.37 ± 1.35 N (BF-10). Similar results were reported by Barbut [92], who highlighted the increase in hardness of lean poultry meat paste supplemented with plant fibers, from 34.6 ± 1. 18 N in control sample to 36.09 ± 0.98 N with apple fiber addition and 41.09 ± 1.99 N with sugarcane fiber addition. On the other hand, Choi et al. [23], demonstrated that fat substitution with chicory fiber significantly decreased the hardness of restructured sausages from 0.56 kg in the control sample to 0.40 kg for the formulation with 10% chicory fiber.

Cohesiveness values varied from 0.41 ± 0.01 to 0.54 ± 0.03, with low variability between samples, suggesting that fiber additions did not significantly alter the dough’s ability to remain compact during deformation. These results are consistent with a study conducted by Ağar et al. [89] which reported increased cohesiveness in meat emulsions enriched with sugar beet fiber, from 0.494 ± 0.00 to a value of 0.593 ± 0.01 for the maximum level of addition. Barbut [92] also reported that the addition of 2% apple fiber in poultry meat batters increased the cohesiveness from 0.35 ± 0.01 in the control sample to 0.37 ± 0.01.

Elasticity was lowest in the control (0.47 ± 0.03) but increased significantly at 3% inclusion for both fibers, before stabilizing near control values at higher levels. The higher elasticity observed at a 3% addition reflects the optimal interaction between fibers and proteins, favoring the formation of a well-hydrated and elastic network [35]. At this level, the fibers complement the internal matrix of the product without destabilizing it, while higher amounts of fibers (6% and 10%) affect the availability of water for protein hydration, reducing their ability to form a uniform elastic network [91,93]. Similar observations in terms of the elastic properties were reported by Younis and Ahmad (2015) [56], who showed an increase in springiness when incorporating 6% apple pomace powder into buffalo meat sausage (from 0.78 ± 0.02 to 0.81 ± 0.01 mm), suggesting the effect of dietary fibers to form a to jelly structure during processing. In contrast, Ağar et al. [89] determined that the addition of sugar beet fiber caused a decrease in springiness of meat emulsions from an initial value of 0.952 ± 0.01 to 0.866 ± 0.04 at the highest addition level.

Gumminess increased significantly (*p* < 0.05) with fiber concentration, especially in the case of beet fiber, where a maximum value of 12.36 ± 0.73 N was recorded at a 10% addition, consistent with its higher hardness and cohesiveness. Adhesiveness decreased at addition levels of 6% and 10% for both fiber types, compared with the control and 3% variants. Notably, the 3% apple fiber sample showed a significantly (*p* < 0.05) higher adhesiveness value (0.42 ± 0.52 mJ), suggesting stronger water retention and enhanced fiber–protein bonding. Barbut [92] reported similar results of increasing gumminess from 11.91 ± 0.49 in control meat batters to 13.75 ± 0.70 in meat batters enriched with apple fiber. Similarly, Ağar et al. [89] observed elevated gumminess and chewiness in chicken sausages enriched with quinoa flour, attributed to enhanced water entrapment.

Overall, the results obtained for texture profile analysis parameters are consistent with previous reports indicating that the incorporation of dietary fibers into emulsified or comminuted meat products reinforces texture parameters. Naghdi et al. [42] studied the effect of addition of quinoa flour and date seed powder on the physicochemical properties of chicken and fish nuggets. The results of the textural analysis showed significant higher hardness and gumminess for the added treatments compared to the control treatment. Additionally, a study adding apple fiber and papain in restructured meat loaf showed a significant increase in firmness by forming a rigid and cohesive structure of the meat loaves formulated with papain and fiber compared to the control treatment [34]. These researchers attributed the texture modifications of these treatments to the lower concentration of water, the dietary fibers’ water holding capacity and its ability to form a cohesive matrix structure. Furthermore, adding 6% apple pomace powder to buffalo meat sausage resulted in a significant increase in firmness, springiness and cohesiveness property of the final product, compared to the control sausage Younis & Ahmad [56]. These results support the textural trends observed in the present study and confirm that fiber–protein interactions play a major role in determining mechanical resistance in restructured meat systems.

### 3.5. Sensory Perception and Evaluation Results

**Figure 2:**
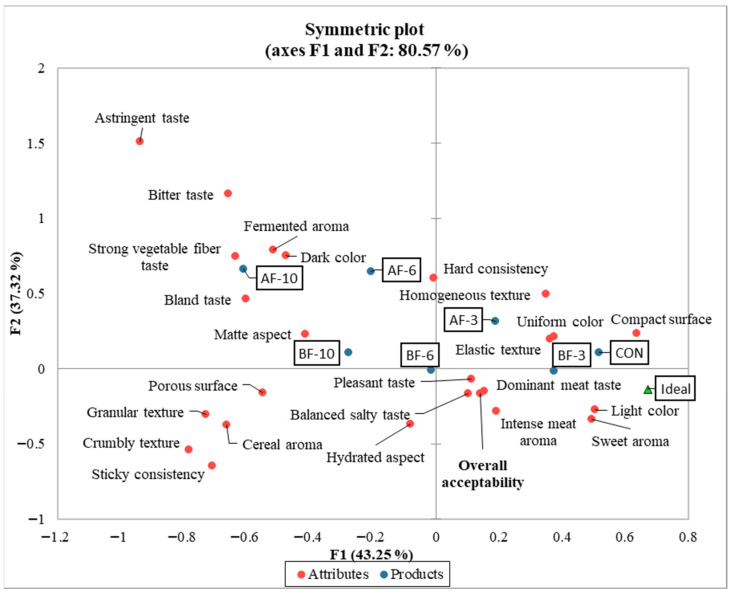
Graphical representation (symmetric plot) of CATA analysis for the seven samples of meat pasta with dietary fiber.

**Figure 3:**
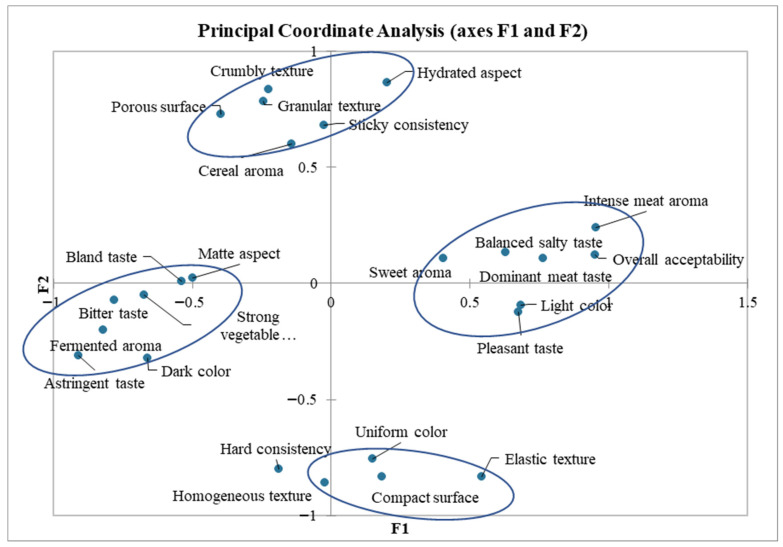
Principal coordinate analysis (PCoA) of the relationships between CATA attributes.

The CATA (Check-all-that-apply) test involves evaluating products by checking the attributes in the questionnaire that the evaluator perceives as present in the analyzed sample, while simultaneously comparing the experimental samples with an ideal product, whose characteristics have been previously established. Figure 2 and Figure 3 illustrate the graphical results of applying CATA statistical test to the data collected from the sensory questionnaires. The graphs visually represent the relationships between the sensory attributes that were selected simultaneously to describe a product.

The symmetric plot (Figure 2) illustrates the distribution of the evaluated products according to the relevant sensory attributes following the application of the Check-all-that-apply (CATA) questionnaire. By positioning both the samples and the sensory attributes, the plot enables clear interpretation and visualization of the relationships between them. The main axes F1 (43.25%) and F2 (37.32%) explain 80.57% of the total data variability, indicating a good representation of the sensory differentiation among the analyzed products. The placement of the ideal on the graph provides a benchmark for the sensory characteristics most preferred by the evaluators and highlights the experimental variants perceived as most similar to this ideal.

Based on samples positioning, the variants closest to the ideal are the control and BF-3 (with 3% beet fiber), suggesting that they were the most balanced from a sensory perspective. Furthermore, the samples located in the right quadrant (the control and the variants with the lowest addition level—3%), delimited by the vertical axis F1, were characterized by positive sensory attributes such as dominant meat taste, balanced salty taste, pleasant taste, intense meat aroma, light color, uniform color, and sweet aroma.

The horizontal axis F2 separates attributes considered to represent taste and color defects (upper left quadrant), while also grouped batches AF-6, AF-10, and BF-10. Samples AF-6 and AF-10 were positioned in the area dominated by negative sensory characteristics, such as bitter taste, astringent taste, fermented aroma, pronounced taste of vegetable fibers, and dark color. These products being perceived less favorably, most likely due to oxidative processes. These findings suggest that apple fiber exerted the most pronounced influence on the product, and this effect is also reflected in consumer perception.

The principal coordinate analysis (PCoA) presented in Figure 3 illustrates related sensory characteristics; more specifically, those clustered within the same circle indicate attributes that evaluators most frequently checked together. The positioning of attributes in this graph highlights their correlations. Attributes located close together, within the same clusters, are perceived as being frequently associated with the same product category. Overall acceptability, as a general sensory attribute based on hedonic evaluation, was positioned in relation to the sensory characteristics that contributed most to the highest hedonic score.

The PCoA chart displays four groups of sensory attributes, with the most favorable ones, simultaneously selected by the evaluators, positioned on the right, close to the horizontal axis F2. These include attributes such as pleasant taste, balanced salty taste, dominant meat taste, intense meat aroma, sweet aroma, and light color. According to Figure 2, the control sample and BF-3 are the most strongly positively correlated with these attributes. In the lower region of the vertical axis F1, another group of attributes—elastic texture, homogeneous, compact surface, uniform color—were positively correlated by evaluators. Based on the CATA graph (Figure 2), these attributes best describe the samples with minimum levels of additives (AF-3 and BF-3) and the control sample.

The group of undesirable sensory attributes, positioned on the left side of the graph, included taste, aroma, and appearance defects, such as bitter taste, bland taste, pronounced vegetable fiber taste, fermented aroma, astringent taste, dark color, and dull appearance. These descriptors are generally associated with oxidative processes or an imbalance in taste properties caused by additives perceived negatively by consumers.

Texture-related defects such as crumbly or grainy texture, porous surface, sticky consistency, and cereal aroma, which indicate structural problems caused by excessive fiber addition or high-water absorption during boiling, were not identified in high proportions. Although positively associated as descriptors, these attributes were largely unrelated to the pasta samples.

Quantitative descriptive analysis (QDA) was performed to evaluate the intensity of each sensory attribute identified in the CATA test, using the 9-point hedonic scale. Each sensory attribute was rated by the evaluators from 1 (not characteristic) and 9 (strongly characteristic of the sample). The sensory questionnaire results were statistically analyzed using ANOVA with Levene’s post hoc test to assess the effects of variation factors (fiber type and percentage of addition) on sensory perception.

The results presented in Table 6 show that the addition of different fiber types and concentrations significantly (*p* < 0.05) influenced most sensory attributes. Color-related attributes such as light color, dark color, and uniform color varied significantly (*p* < 0.05), with the control sample (Con) recording the highest scores for uniformity and light color. Conversely, samples with higher fiber inclusion (10%) tended to be darker and less uniform. For these color-related sensory attributes the level of fiber addition appeared to have a greater influence compared to the fiber type.

The fiber addition significantly affected taste and aroma attributes (*p* < 0.001). The control (Con) and the 3% addition formulations were associated with higher acceptability and a well-balanced meat taste. However, depending on the fiber type, specific aromas were perceived, especially at high concentrations. For example, samples enriched with apple fiber had fermented notes and a slightly bitter taste, which may negatively affect sensory acceptability.

In terms of textural attributes, significant differences (*p* < 0.001) were observed for elastic, granular, and homogeneous texture, and also for firmness, indicating that fiber inclusion modified the textural properties of the meat pasta. Notably, the apple fiber variants were described by the evaluators as having greater elasticity and a smoother, more homogeneous structure. A certain degree of score dispersion was observed for sensory attributes, which is typical and well-documented in descriptive sensory analysis [94,95], as individual perceptual sensitivity, prior experience, and threshold recognition can influence attribute scoring.

To provide deeper insight into consumer sensory perception, four general attributes from the QDA evaluation—uniform color, pleasant taste, homogeneous texture, and overall acceptability—were selected for graphical representation. These attributes are widely recognized as determinants of sensory quality and purchase intention, as they integrate multimodal cues that guide rapid hedonic judgment. Color, for example, is one of the strongest visual indicators of product freshness and quality, shaping expectations regarding flavor and texture and modulating consumer behavior [83]. Texture has been identified as a major driver of both acceptance and rejection, influencing eating behavior, mouthfeel perception, and product familiarity [96]. Furthermore, the balance between flavor, color, and texture must be co-optimized to maximize consumer acceptance, particularly when functional ingredients such as dietary fibers are incorporated [35,86].

**Figure 4:**
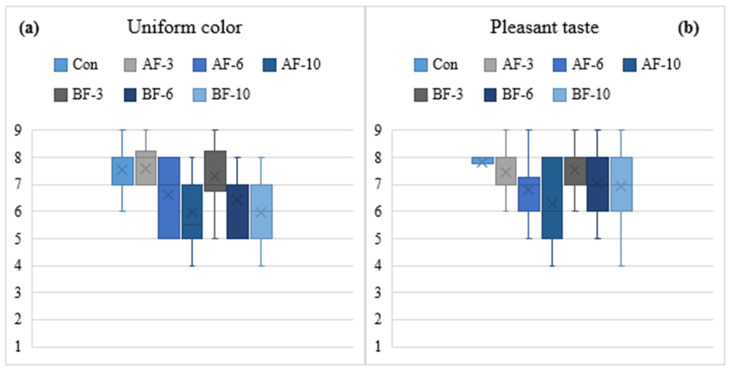
Boxplot showing the distribution of panelists’ scores for (**a**) uniform color and (**b**) pleasant taste across meat pasta samples with added dietary fibers (Con—control sample, AF—apple fiber, BF—sugar beet fiber at different inclusion levels—3%, 6%, 10%). Boxes represent the interquartile range, lines indicate minimum and maximum values (whiskers), and “×” marks the mean score.

The boxplot analysis of uniform color (Figure 4a) revealed that samples containing added fiber at moderate levels (AF-3 and BF-3), along with the control exhibited higher median scores and narrower variability compared to the other formulations, suggesting a more stable and visually acceptable color profile. Djordjević et al. [97] indicate in a previous study that moderate vegetable fiber incorporation can preserve lightness and reduce irregular surface pigmentation, supporting consumer expectations of familiar meat-based products. Pleasant taste scores were highest in the control and AF-3/BF-3 samples, whereas AF-6 and AF-10 exhibited lower medians and wider heterogeneity. These findings suggest negative taste interactions at elevated apple fiber concentrations, potentially associated with reducing the characteristic flavors of meat, and the presence of fermented or bitter notes. Similar reductions in consumer liking at high fiber contents have been reported in fiber-enriched foods [34,98], confirming the dose-dependent nature of sensory acceptance.

**Figure 5:**
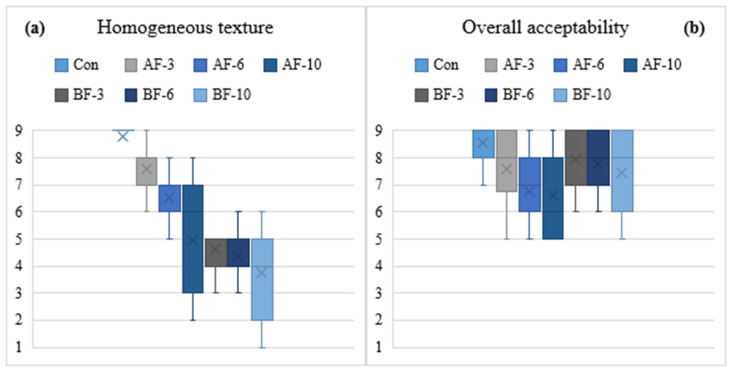
Boxplot showing the distribution of panelists’ scores for (**a**) Homogeneous texture (**b**) Overall acceptability across meat pasta samples with added dietary fibers (Con—control sample, AF—apple fiber, BF—sugar beet fiber at different inclusion levels—3%, 6%, 10%). Boxes represent the interquartile range, lines indicate minimum and maximum values (whiskers), and “×” marks the mean score.

As shown in Figure 5a, scores for texture homogeneity decreased with increasing fiber addition, especially in AF-10, reflecting matrix densification and structural heterogeneity. Soluble fruit fibers are known to bind water strongly, producing denser, less cohesive textures at high concentrations [99]. Conversely, moderate sugar-beet fiber promoted a more elastic and uniform network, consistent with previous study reported by Ağar et al. [89], demonstrating the use of sugar beet fiber as a good alternative of enriching low-fat frankfurters with dietary fiber, without significantly affecting the sensory score of the product. Overall acceptability plots (Figure 5b) showed that the control and samples containing sugar beet fiber (BF-3%, BF-6%) exhibited higher median values and narrower score distributions, indicating a better perceived overall quality by the panelists. In contrast, samples with apple fiber content, and also with a higher sugar beet fiber (BF-10%) presented lower medians and wider variability, suggesting reduced consumer acceptability and greater heterogeneity in perception, with some panelists rejecting these samples more strongly than others.

Overall, the quantitative descriptive analysis showed that fiber type and concentration, as well as the interaction between these factors, significantly (*p* < 0.05) influenced the sensory profile of meat pasta, affecting color, texture, taste, and overall acceptability. While low fiber concentrations maintained a favorable sensory profile, higher fiber levels led to a more pronounced perception of undesirable sensory attributes.

## 4. Conclusions

The present study demonstrated that the incorporation of apple and sugar beet fibers into meat-based pasta formulations significantly influenced their chemical composition, particularly fiber content, protein and fat distribution, due to fiber–protein and fiber–water interactions, color and textural parameters, and also sensory properties. From a nutritional perspective, the addition of fruit-derived fibers represents an efficient strategy to increase the dietary fiber content of traditional meat products, thereby addressing consumer demands for healthier and more functional foods. The enriched pasta formulations improved the nutritional profile, while also maintaining satisfactory sensory acceptance, particularly at moderate fiber levels, where panelists appreciated the enhanced firmness and structural uniformity.

Textural analysis revealed that apple fiber, rich in pectin, contributed to the formation of a well-hydrated, elastic network at moderate inclusion levels (3%), while sugar beet fiber, characterized by its high water-binding capacity, promoted firmness and structural density. On the other hand, excessive supplementation (6–10%) negatively affected elasticity and cohesiveness, suggesting that high fiber concentrations may compete with proteins for water availability and disrupt the uniformity of the protein matrix. However, these improvements must be interpreted in relation to sensory acceptance, as higher inclusion levels (6–10%) led to darker coloration, fermented and astringent notes, and a denser, less homogeneous structure, characteristics commonly associated with lower consumer liking in meat products. This trade-off highlights that maximizing nutritional enhancement may negatively affect perceived quality attributes, reinforcing the importance of identifying a balanced inclusion level that maintains technological functionality without compromising sensory appeal.

Beyond the nutritional improvements, the use of apple and sugar beet fibers contributes to a broader sustainability framework. Both raw materials are frequently obtained as by-products of the fruit juice and sugar industries, and their incorporation into meat-based products supports the principles of the circular economy by upcycling these underutilized materials into value-added food ingredients. Overall, this study provides evidence that moderate fiber supplementation (3%) is promising for producing innovative, nutritious, and appealing meat-based pasta.

A direction for future research is to focus on the presence of naturally occurring bioactive compounds in apple and beet fibers (such as polyphenols, minerals, and antioxidant pigments) that can contribute to the functional potential of the enriched formulations, and also investigate shelf-life behavior, oxidative stability, and microbiological safety under refrigerated storage.

## Figures and Tables

**Table 1:** Recipe for meat pasta production (reported per 100 g).

Ingredients	Amount (g)						
	CON	AF-3	AF-6	AF-10	BF-3	BF-6	BF-10
Pork leg meat	57	57	57	57	57	57	57
Semolina flour	33	30	27	23	30	27	23
Apple fiber	0	3	6	10	0	0	0
Sugar beet fiber	0	0	0	0	3	6	10
Salt	2.4	2.4	2.4	2.4	2.4	2.4	2.4
Spices	2.1	2.1	2.1	2.1	2.1	2.1	2.1
Bone broth	5.5	5.5	5.5	5.5	5.5	5.5	5.5
Total	100	100	100	100	100	100	100

**Table 2:** Sensory attributes assessed by the evaluators.

Sensory Characteristics	Sensory Terms
Appearance	light color; dark color; uniform color; matte aspect; hydrated aspect; compact surface; porous surface.
Aroma	intense meat aroma; sweet aroma; cereal aroma; fermented aroma.
Taste	balanced salty taste; bitter taste; pleasant taste; dominant meat taste; astringent taste; strong vegetable fiber taste; bland taste.
Texture	elastic texture; granular texture; homogeneous texture; sticky consistency; crumbly texture; hard consistency.
Overall impression	overall acceptability.

**Table 3:** Chemical composition of the control and fiber enriched meat pasta.

Chemical Characteristics	CON	AF-3	AF-6	AF-10	BF-3	BF-6	BF-10
Dry matter	74.80 ± 0.16 ^aA^	66.01 ± 0.89 ^cC^	65.65 ± 0.28 ^cB^	70.05 ± 0.07 ^bA^	68.32 ± 0.29 ^bB^	64.72 ± 0.20 ^dB^	67.85 ± 0.09 ^cB^
Humidity	25.20 ± 0.16 ^dC^	33.99 ± 0.89 ^aA^	34.35 ± 0.28 ^aA^	29.95 ± 0.07 ^bB^	31.68 ± 0.28 ^cB^	35.28 ± 0.20 ^aA^	32.15 ± 0.09 ^bA^
Fat	2.06 ± 0.10 ^bB^	2.23 ± 0.10 ^bA^	2.24 ± 0.14 ^bA^	2.78 ± 0.12 ^aA^	2.16 ± 0.08 ^abAB^	2.27 ± 0.06 ^aA^	2.30 ± 0. 10 ^aB^
Total protein	23.82 ± 0.13 ^aA^	21.52 ± 0.27 ^cC^	20.61 ± 0.17 ^dA^	22.07 ± 0.08 ^bA^	22.26 ± 0.09 ^bB^	19.68 ± 0.11 ^dB^	20.93 ± 0.07 ^cC^
Ash	4.88 ± 0.04 ^aA^	4.44 ± 0.06 ^bC^	4.22 ± 0.03 ^cB^	4.42 ± 0.02 ^bB^	4.66 ± 0.03 ^cB^	4.69 ± 0.01 ^cA^	5.02 ± 0.03 ^aA^
Total fiber	2.94 ± 0.01 ^dC^	5.02 ± 0.10 ^cA^	7.36 ± 0.12 ^bA^	11.15 ± 0.06 ^aA^	4.77 ± 0.07 ^cB^	6.49 ± 0.06 ^bB^	9.59 ± 0.08 ^aB^
Carbohydrates	41.11 ± 0.15 ^aA^	32.80 ± 0.57 ^bC^	31.22 ± 0.19 ^cB^	29.63 ± 0.20 ^dB^	34.46 ± 0.20 ^bB^	31.59 ± 0.16 ^cB^	30.00 ± 0.13 ^dB^
Energy value (kcal 100 g^−1^)	284.11 ± 0.91 ^aA^	247.62 ± 3.30 ^cC^	242.42 ± 1.52 ^dB^	254.82 ± 1.00 ^bB^	255.92 ± 1.35 ^bB^	238.50 ± 0.77 ^dC^	243.64 ± 0.56 ^cC^

The average ± standard error values, different letters “^A^”, “^B^”, “^C^” in the rows indicate statistically significant differences (*p* < 0.05) between the fiber types with the same percentage of addition and “^a^”, “^b^”, “^c^”, “^d^” in the rows indicate statistically significant differences (*p* < 0.05) between different percentages of addition (same fiber type).

**Table 4:** Color parameters of meat pasta after drying.

Color	Control	Apple Fiber			Sugar Beet Fiber		
Parameters	Con	AF-3	AF-6	AF-10	BF-3	BF-6	BF-10
L*	42.68 ± 1.72 ^b^	44.68 ± 2.13 ^b^	34.99 ± 1.62 ^c^	36.46 ± 2.45 ^c^	48.83 ± 1.27 ^a^	50.42 ± 1.52 ^a^	50.67 ± 1.53 ^a^
a*	13.63 ± 0.78 ^a^	7.05 ± 0.99 ^c^	6.88 ± 0.25 ^c^	5.21 ± 0.51 ^d^	8.49 ± 0.47 ^b^	7.55 ± 0.29 ^bc^	7.58 ± 0.45 ^bc^
b*	9.31 ± 0.82 ^c^	10.44 ± 0.83 ^c^	7.69 ± 0.66 ^d^	5.91 ± 0.31 ^e^	14.10 ± 0.85 ^a^	14.75 ± 0.70 ^a^	12.75 ± 0.23 ^b^
C* (Chroma)	16.51 ± 1.08 ^a^	12.61 ± 1.15 ^c^	10.33 ± 0.47 ^d^	7.88 ± 0.56 ^e^	16.46 ± 0.95 ^a^	16.57 ± 0.73 ^a^	14.83 ± 0.33 ^b^
H* (Hue angle)	0.60 ± 0.02 ^d^	0.98 ± 0.05 ^b^	0.84 ± 0.05 ^c^	0.85 ± 0.03 ^c^	1.03 ± 0.01 ^b^	1.10 ± 0.01 ^a^	1.03 ± 0.03 ^b^

L*: lightness from 100 for perfect white to zero of black. a*: + redness, − greenness. b*: + yellowness, − blueness. The average ± standard error values, same superscript letters indicate no significant difference, and different letters indicate a significant difference in each row.

**Table 5:** Descriptive parameters for the texture of meat pasta.

Parameters	Sample	CON	AF-3	AF-6	AF-10	BF-3	BF-6	BF-10
Warner–Bratzler shear force, N/cm^2^	Uncooked pasta	4.82 ± 0.68 ^cd^	6.14 ± 0.22 ^b^	5.38 ± 0.60 ^bcd^	7.57 ± 0.53 ^a^	4.27 ± 0.09 ^d^	5.82 ± 1.04 ^bc^	6.48 ± 0.35 ^ab^
	Cooked pasta	1.20 ± 0.14 ^ab^	1.01 ± 0.13 ^ab^	1.18 ± 0.20 ^ab^	0.91 ± 0.11 ^b^	1.32 ± 0.17 ^a^	1.23 ± 0.20 ^ab^	1.17 ± 0.19 ^ab^
Work of shear, mJ	Uncooked pasta	37.77 ± 1.34 ^cd^	42.88 ± 3.05 ^bcd^	46.28 ± 1.37 ^bc^	47.43 ± 5.73 ^b^	36.70 ± 1.55 ^d^	58.84 ± 2.15 ^a^	48.58 ± 9.20 ^b^
	Cooked pasta	18.19 ± 3.14 ^a^	14.23 ± 1.91 ^ab^	14.69 ± 1.06 ^ab^	13.68 ± 2.31 ^ab^	15.27 ± 1.99 ^ab^	12.90 ± 1.83 ^c^	11.64 ± 1.91 ^b^
Hardness, N	Uncooked pasta	8.01 ± 0.42 ^e^	14.24 ± 0.38 ^d^	12.68 ± 0.72 ^d^	21.23 ± 0.57 ^b^	13.74 ± 1.37 ^d^	18.13 ± 2.19 ^c^	26.37 ± 1.35 ^a^
Cohesiveness	Uncooked pasta	0.41 ± 0.01 ^c^	0.47 ± 0.02 ^abc^	0.47 ± 0.03 ^abc^	0.49 ± 0.02 ^ab^	0.51 ± 0.04 ^a^	0.45 ± 0.03 ^bc^	0.47 ± 0.05 ^abc^
Gumminess, N	Uncooked pasta	3.28 ± 0.24 ^f^	6.71 ± 0.38 ^de^	5.92 ± 0.67 ^e^	10.43 ± 0.54 ^b^	7.02 ± 0.30 ^cd^	8.02 ± 0.47 ^c^	12.36 ± 0.73 ^a^
Springiness	Uncooked pasta	0.47 ± 0.03 ^b^	0.53 ± 0.02 ^ab^	0.49 ± 0.02 ^b^	0.49 ± 0.01 ^b^	0.77 ± 0.08 ^a^	0.55 ± 0.34 ^ab^	0.47 ± 0.04 ^b^
Adhesiveness, mJ	Uncooked pasta	0.21 ± 0.04 ^b^	0.42 ± 0.52 ^a^	0.08 ± 0.09 ^b^	0.06 ± 0.02 ^b^	0.28 ± 0.10 ^b^	0.06 ± 0.02 ^b^	0.07 ± 0.05 ^b^

The average ± standard error values, common letters indicate no significant difference, and different letters indicate a significant difference in each column.

**Table 6:** Mean ± standard deviation for the sensory characteristics of meat pasta samples with added fiber.

Sensory Attribute	Control	Apple Fiber			Sugar Beet Fiber			*p*-Value		
	Con	AF-3	AF-6	AF-10	BF-3	BF-6	BF-10	F1	F2	F1*F2
Light color	7.17 ± 1.12	4.90 ± 0.88	4.23 ± 0.73	3.43 ± 1.50	7.30 ± 0.92	6.77 ± 1.10	6.17 ± 1.29	**0.000**	**0.000**	0.708
Dark color	4.80 ± 0.71	6.90 ± 1.18	7.40 ± 0.93	7.73 ± 1.11	4.37 ± 1.03	4.50 ± 1.31	4.73 ± 1.34	**0.000**	**0.013**	0.479
Uniform color	7.53 ± 0.94	7.57 ± 1.33	6.63 ± 1.22	5.97 ± 1.19	7.30 ± 1.32	6.43 ± 1.14	6.00 ± 1.34	0.427	**0.000**	0.778
Matte aspect	1.70 ± 1.02	3.97 ± 1.10	4.10 ± 0.99	5.13 ± 1.31	2.83 ± 0.87	4.67 ± 1.21	4.80 ± 1.19	0.071	**0.000**	**0.000**
Hydrated aspect	4.53 ± 1.04	2.03 ± 1.22	1.97 ± 1.13	2.10 ± 1.06	5.10 ± 1.30	5.83 ± 1.05	5.93 ± 1.26	**0.000**	0.089	0.103
Compact surface	7.57 ± 1.04	6.13 ± 1.43	5.73 ± 1.36	4.77 ± 0.86	6.80 ± 1.27	6.03 ± 1.52	5.03 ± 0.49	**0.021**	**0.000**	0.594
Porous surface	2.83 ± 0.87	2.97 ± 1.07	3.03 ± 1.97	3.90 ± 1.45	2.93 ± 1.05	3.43 ± 1.01	2.77 ± 1.57	0.200	0.266	**0.006**
Intense meat aroma	7.20 ± 0.92	6.43 ± 1.76	4.30 ± 1.24	3.73 ± 1.64	6.93 ± 0.83	6.33 ± 1.37	6.17 ± 1.29	**0.000**	**0.000**	**0.000**
Sweet aroma	3.47 ± 1.17	1.93 ± 1.23	1.60 ± 1.04	1.73 ± 1.01	3.63 ± 1.35	3.23 ± 1.38	2.53 ± 1.72	**0.012**	0.076	0.505
Cereal aroma	1.00 ± 0.00	1.47 ± 1.17	1.57 ± 0.73	1.63 ± 0.61	1.07 ± 0.25	1.20 ± 0.76	1.53 ± 1.11	**0.000**	**0.023**	0.107
Fermented aroma	1.00 ± 0.00	2.73 ± 0.83	5.20 ± 1.52	5.23 ± 1.48	1.93 ± 1.11	2.77 ± 1.57	2.80 ± 1.54	**0.000**	**0.000**	**0.000**
Balanced salty taste	6.43 ± 1.98	6.20 ± 0.66	4.93 ± 1.41	4.77 ± 1.45	6.17 ± 1.78	5.93 ± 1.46	5.80 ± 1.32	**0.003**	**0.002**	0.086
Bitter taste	1.10 ± 0.55	1.93 ± 0.78	2.57 ± 1.55	3.03 ± 1.13	1.23 ± 0.63	1.37 ± 0.93	1.40 ± 0.86	**0.000**	**0.002**	**0.032**
Pleasant taste	7.83 ± 0.91	7.47 ± 0.90	6.80 ± 1.16	6.30 ± 1.39	7.53 ± 0.82	7.03 ± 1.33	6.93 ± 1.31	0.068	**0.000**	0.376
Dominant meat taste	8.63 ± 0.67	7.67 ± 1.27	6.33 ± 0.61	5.87 ± 1.31	7.73 ± 1.28	7.27 ± 1.11	6.90 ± 1.18	**0.000**	**0.000**	**0.031**
Astringent taste	1.00 ± 0.00	1.80 ± 1.16	4.40 ± 1.43	4.73 ± 1.41	1.10 ± 0.31	1.17 ± 0.38	1.20 ± 0.41	**0.000**	**0.000**	**0.000**
Strong fiber taste	1.00 ± 0.00	2.03 ± 1.47	1.57 ± 1.14	2.63 ± 2.20	1.37 ± 0.81	1.43 ± 0.77	1.53 ± 0.94	**0.001**	**0.031**	0.097
Bland taste	1.00 ± 0.00	1.70 ± 1.15	2.47 ± 1.55	2.43 ± 1.28	1.43 ± 0.68	2.20 ± 1.16	2.77 ± 1.57	0.703	**0.000**	0.272
Elastic texture	6.93 ± 0.94	7.27 ± 0.91	6.83 ± 1.32	6.22 ± 1.55	7.63 ± 0.93	7.13 ± 1.48	6.73 ± 1.46	**0.029**	**0.000**	0.896
Granular texture	1.27 ± 0.45	2.10 ± 1.16	5.13 ± 1.33	5.17 ± 1.49	3.50 ± 1.22	5.73 ± 1.36	5.90 ± 1.18	**0.000**	**0.000**	0.156
Homogeneous texture	8.80 ± 0.61	7.60 ± 0.86	6.53 ± 0.78	4.93 ± 2.02	4.63 ± 1.35	4.37 ± 1.16	3.77 ± 1.61	**0.000**	**0.000**	**0.001**
Sticky consistency	3.40 ± 0.77	3.47 ± 2.50	3.63 ± 1.25	3.27 ± 1.05	3.53 ± 0.73	3.63 ± 0.72	3.67 ± 1.30	0.431	0.766	0.675
Crumbly texture	1.67 ± 1.09	1.93 ± 1.14	3.67 ± 1.30	4.47 ± 1.43	1.97 ± 1.30	3.43 ± 1.50	3.87 ± 1.46	0.179	**0.000**	0.423
Hard consistency	6.67 ± 1.30	6.80 ± 1.19	6.07 ± 1.78	5.97 ± 1.19	6.90 ± 1.16	5.83 ± 1.49	7.17 ± 0.95	0.071	**0.001**	0.009
Overall acceptability	8.57 ± 0.73	7.60 ± 1.30	6.77 ± 1.30	6.60 ± 1.35	7.97 ± 1.13	7.77 ± 1.04	7.47 ± 1.33	**0.000**	**0.002**	0.308

F1—fiber type; F2—fiber concentration; ANOVA and Levene’s post hoc test were used to examine differences between groups due to the two factors of variation or their interaction. Significant differences between groups are indicated by *p* < 0.05.

## Data Availability

The original contributions presented in the study are included in the article, further inquiries can be directed to the corresponding authors.
